# Nasal immunization with recombinant chimeric pneumococcal protein and cell wall from immunobiotic bacteria improve resistance of infant mice to *Streptococcus pneumoniae* infection

**DOI:** 10.1371/journal.pone.0206661

**Published:** 2018-11-05

**Authors:** Jonathan Laiño, Julio Villena, Alexander Suvorov, Hortensia Zelaya, Ramiro Ortiz Moyano, Susana Salva, Susana Alvarez

**Affiliations:** 1 Laboratory of Immunobiotechnology, Reference Centre for Lactobacilli (CERELA-CONICET), Tucuman, Argentina; 2 Federal State Budgetary Scientific Institution “Institute of Experimental Medicine”, Saint-Petersburg, Russia; 3 Saint-Petersburg State University, Saint-Petersburg, Russia; Instituto Butantan, BRAZIL

## Abstract

Respiratory tract infections and invasive disease caused by *Streptococcus pneumoniae* in high-risk groups are a major global health problem. Available human vaccines have reduced immunogenicity and low immunological memory in these populations, as well as high cost as a public health strategy in poor communities. In addition, no single pneumococcal protein antigen has been able to elicit protection comparable to that achieved using protein-polysaccharide conjugate vaccines. In this context, chimeric pneumococcal proteins raise as potential good vaccine candidates because of their simplicity of production and reduced cost. The aim of this work was to study whether the nasal immunization of infant mice with the recombinant chimeric pneumococcal protein (PSFP) was able to improve resistance to *S*. *pneumoniae*, and whether the immunomodulatory strain *Lactobacillus rhamnosus* CRL1505 or its cell wall (CW1505) could be used as effective mucosal adjuvants. Our results showed that the nasal immunization with PSPF improved pneumococcal-specific IgA and IgG levels in broncho-alveolar lavage (BAL), reduced lung bacterial counts, and avoided dissemination of pneumococci into the blood. Of interest, immunization with PSPF elicited cross-protective immunity against different pneumococcal serotypes. It was also observed that the nasal immunization of infant mice with PSPF+CW1505 significantly increased the production of pneumococcal-specific IgA and IgG in BAL, as well as IgM and IgG in serum when compared with PSPF alone. PSPF+CW1505 immunization also improved the reduction of pneumococcal lung colonization and its dissemination in to the bloodstream when compared to PSPF alone. Our results suggest that immunization with PSPF together with the cell wall of the immunomodulatory strain *L*. *rhamnosus* CRL1505 as a mucosal adjuvant could be an interesting alternative to improve protection against pneumococcal infection in children.

## Introduction

*Streptococcus pneumoniae* (the pneumococcus) is a major global health problem since it is a common cause of respiratory tract infections and invasive disease especially in high-risk groups like children in their first few years of life, elderly and immunocompromised patients. The emergence of multiantibiotic-resistant pneumococcal strains throughout the world has led to an increased emphasis on the prevention of *S*. *pneumoniae* infections by vaccination [1,2]. However, vaccines available for application in human still have disadvantages associated with their reduced immunogenicity and low immunological memory in high-risk populations like children (i.e., pneumococcal polysaccharide vaccines) or with their high cost as a public health strategy in poor communities (i.e., conjugated vaccines) [3]. Therefore, current efforts in pneumococcal vaccine development are focused on exploring alternative strategies able to address the shortcomings of existing formulations.

The most studied strategy in the development of new effective pneumococcal vaccines involves the use of one or more pneumococcal protein antigens common to all serotypes. These vaccines are able to confer non-serotype-dependent protection and represent a more economical approach to reduce pneumococcal infections since they can be produced in high-levels with relatively low cost and therefore, they could be more affordable for developing countries. In this regard, virulence factors of protein nature have been studied on the cell surface of *S*. *pneumoniae* [4,5] and several of them have been proved immunogenic and able to induce protection against pneumococcal infection in animal models [3]. However, to date, no single pneumococcal protein antigen has been able to elicit protection comparable to that achieved using protein-polysaccharide conjugate vaccines.

In order to improve the efficiency of pneumococcal protein vaccines, it would be necessary to include different antigens in the formulation to cover the most important virulence determinants present in all serotypes [6,7]. However, the formulation of vaccines including separate antigenic proteins makes the vaccine a lot more difficult for standardization and it significantly enhance the cost. As an alternative, scientists have considered chimeric pneumococcal proteins as vaccine candidates [8,9] because of their simplicity of production and reduced cost effectiveness compared with vaccines made by mixtures of protein antigens.

In a previous study, we developed a chimeric protein composed of the fragments of three surface proteins of *S*. *pneumoniae*: pneumococcal surface adhesion A (PsaA), pneumococcal surface protein A (PspA) and the surface protein Spr1875 [10]. Taking into consideration that flagellin from Gram-negative bacteria is able to interact with Toll-like receptor-5 (TLR-5) and therefore to stimulate the innate immune system [11], the fragments FliC from *Salmonella typhimurium* flagellin were included into the chimeric pneumococcal protein as adjuvants [10]. The molecular design of the artificial chimeric protein was performed *in silico* according to the immunogenic domains and putative three-dimensional structures of PsaA, Spr1875, PspA, and FliC fragments, separated with appropriate flexible bridges [10]. The recombinant chimeric pneumococcal protein with a total length of 560 amino acids was produced in *E*. *coli* and was named PSPF (PsaA- Spr1875-PspA-FliC).

The immunogenicity and protective capacity of PSPF was tested in adult immunocompetent BALB/c mice and it was demonstrated that subcutaneous immunization improved resistance of animals to intraperitoneal and nasal challenges with virulent pneumococcal serotypes [10]. However, the efficacy of mucosally administered PSPF to confer protective immunity or its effect in a high-risk population has not been evaluated. Therefore, in this work we aimed to study whether the nasal immunization of infant Swiss albino mice, which are known to be more susceptible to pneumococcal infection when compared to adult immunocompetent mice [12,13], with PSPF was able to improve resistance to the respiratory pathogen. Moreover, we evaluated whether the immunomodulatory strain *Lactobacillus rhamnosus* CRL1505 or its cell wall could be used as effective mucosal adjuvants for improving the efficiency of PSPF immunization in infant mice.

## Materials and methods

### Microorganisms and microbial cellular fractions

*Lactobacillus rhamnosus* CRL1505 was obtained from the culture collection of CERELA (Tucumán, Argentina). Lactobacilli (10^10^ CFU stored at -70 °C) were activated and cultured for 12 h at 37 °C (final log phase) in Man-Rogosa-Sharpe (MRS) broth culture media. The bacteria were harvested by centrifugation and washed with sterile PBS (0.01 mol/L, pH 7.2).

Capsulated *Streptococcus pneumoniae* serotype 6B and 14 were obtained from the Administración Nacional de Laboratorios e Institutos de Salud (ANLIS, Buenos Aires, Argentina), *S*. *pneumoniae* serotype 3 und 19F were obtained from the culture collection of the Research Institute of Pediatric Infections (Saint Petersburg, Russia). *S*. *pneumoniae* strains were grown in microanaerobiosis in Blood agar (Columbia agar base) at 37 °C for 18 h, and stored in Todd-Hewitt broth at -70 °C at a concentration of 10^10^ CFU. The four pneumococcal strains were isolated from children with pneumonia.

Non-viable *L*. *ramnhosus* CRL1505 (HK1505) was obtained by heating cell suspension (10^9^ CFU/mL) at 100 °C for 10 min. Loss of cell viability was checked by serial dilutions plates, and colony counting after heat treatment.

For obtaining cell wall from *L*. *rhamnosus* CRL1505, the bacterium was grown in MRS broth for three days, performing periodic refills of new medium in order to concentrate the cells. Cell wall from lactobacilli was obtained using the method of Shida et al. [14] with minor modifications [15]. Briefly, the bacterium was washed 3 times with sterile PBS and lyophilized. Then, the cells were resuspended in sterile water (0.1 g/mL) and were lysed by three sonication cycles in an Ultrasonic Homogenizer (Cole Parmer) with cycles of 2.5 min and 70% of amplitude. Ice bath was used throughout all the sonication process. After the three sonication cycles, the rupture of all the bacterial cells was verified by microbiological methods. The wall obtained in this way was delipidated by successive refluxing with methanol, methanol–chloroform (1:1), and chloroform. The delipidated preparation was resuspended in Tris–HCl buffer (pH 7.2 to 7.5) and treated with bovine pancreatic DNAse I (Sigma) (50 μg/mL) and ribonuclease A (Sigma) (100 μg/mL) at 37 °C with stirring for 4 h. The insoluble material was washed with distilled water and lyophilized. The resultant product was used as the cell wall (CW1505) preparation. The process was applied to 1 gram of the bacteria suspended in 3 mL of sterile PBS. At the end of the cell wall obtainment, the mass yield calculation was performed. The yield of cell wall obtaining was calculated taking into consideration the initial mass of the bacteria and the weight of the lyophilized insoluble material obtained at the end of the process. The dose of cell wall used in the treatment of mice (expressed in μg), was calculated considering the weight of 10^8^ bacterial cells (the optimal dose for immunomodulation) and their correspondence in cell wall weight.

### Animals and ethical statement

Three-week-old male Swiss albino mice were obtained from the closed colony at CERELA (Tucumán, Argentina). Animals were housed in plastic cages and environmental conditions were kept constant, in agreement with the standards for animal housing. Animal welfare was in charge of researchers and special staff trained in animal care and handling at CERELA. Animal health and behaviour were monitored twice a day.

Animals were housed individually during the experiments. All efforts were made to minimize the number of animals and their suffering. Animals were euthanized immediately after the time point was reached, 33 days for immunization experiments and 35 days for immunization-infection experiments. No sings of discomfort or pain were observed before mice reached the endpoints. No deaths were observed before mice reached the endpoints.

This study was carried out in strict accordance with the recommendations in the Guide for the Care and Use of Laboratory Animals of the Guidelines for Animal Experimentation of CERELA. The CERELA Institutional Animal Care and Use Committee prospectively approved this research under the protocol BIOT-CRL-18.

### Immunization protocols

Four sets of experiments with different immunization protocols were used in this work. In the first set of experiments, different groups of infant mice received 10 or 20 μg of PSPF by the nasal route or by subcutaneous injection on days 0, 14 and 28. Nasal groups received 25 μL of PBS containing the PSPF (10 or 20 μg) by dropping 12.5 μL into each nostril. Subcutaneous groups received 100 μL of PBS containing the PSPF (10 or 20 μg) by subcutaneous injection in the back of the animal. Mice treated by the nasal or subcutaneous routes with sterile PBS were used as controls. Five days after the last immunization (day 33) serum and broncho-alveolar lavage (BAL) samples were taken for the determination of specific antibodies or mice were challenged with different *S*. *pneumoniae* serotypes as described below.

In the second set of experiments, different groups of infant mice received 20 μg of PSPF together with 10^8^ cells of HK1505 by the nasal route on days 0, 14 and 28. Mice receiving 20 μg of PSPF only were used as controls. Animals received 25 μL of PBS containing the PSPF or PSPF+HK1505 by dropping 12.5 μL into each nostril. Five days after the last immunization (day 33) serum and BAL samples were taken for the determination of specific antibodies or mice were challenged with different *S*. *pneumoniae* serotypes as described below.

In the third set of experiments, different groups of infant mice received 20 μg of PSPF plus 8 μg of CW1505 by the nasal route on days 0, 14 and 28. The dose of CW1505 was used according to previous studies [15]. Mice receiving 20 μg of PSPF only were used as controls. Animals received 25 μL of PBS containing the PSPF or PSPF+CW1505 by dropping 12.5 μL into each nostril. Five days after the last immunization (day 33) serum and BAL samples were taken for the determination of specific antibodies or mice were challenged with different *S*. *pneumoniae* serotypes as described below.

In the last set of experiments animals were immunized with PSPF or a recombinant chimeric pneumococcal protein lacking the FliC fragments. PSPF and the protein called PSP (PsaA- Spr1875-PspA) were used for the immunization of different groups of infant mice that received 20 μg of PSPF or PSP alone or 20 μg of PSPF or PSP plus 8 μg of CW1505 by the nasal route on days 0, 14 and 28. Serum and BAL samples were taken one day after the last immunization (day 29) for the evaluation of cytokines and five days after the last immunization (day 33) for the determination of specific antibodies.

### Serum and BAL antibodies and cytokines

Specific anti-PSPF antibodies (IgA, IgM, and IgG) were determined by ELISA as described previously [10]. In brief, plates were coated with 1.5 μg of PSPF per well overnight at 4°C and blocked with albumin. Appropriate dilutions of the samples [serum 1:20; BAL 1:2] were incubated for 1 h at 37 °C. Peroxidase conjugated anti-mouse IgG, IgA, or IgM antibodies (1:500) (Sigma-Aldrich) were added and incubated for 1 h at 37 °C. The reaction was developed with TMB Substrate Reagent (Sigma-Aldrich). The concentration was measured with reference to standard curves using known amounts of the respective murine Ig (Sigma-Aldrich).

Tumour necrosis factor (TNF)-α, interferon (IFN)-γ, interleukin (IL)-4, and IL-10 concentrations in BAL were measured with commercially available enzyme-linked immunosorbent assay (ELISA) technique kits following the manufacturer’s recommendations (R&D Systems, MN, USA).

### *S*. *pneumoniae* challenges

*S*. *pneumoniae* serotype 3, 6B, 14, and 19F were first grown on blood agar for 18 h. Freshly grown colonies were suspended in Todd Hewitt broth (Oxoid, Cambridge, UK) and incubated overnight at 37 °C. The pathogens were harvested by centrifugation at 3600×g for 10 min, and then washed three times with sterile PBS. Cell density was adjusted to 1×10^9^ CFU/mL. The size of the inoculum in challenge experiments was confirmed by serial dilutions and quantitative subcultures on blood agar.

Mice were challenged nasally with 10^7^ CFU of the pathogen per mouse. Animals were infected by dropping 12.5 μL of *S*. *pneumoniae* in PBS into each nostril, which was then involuntarily inhaled. To facilitate migration of the inoculum to the alveoli, mice were held in a head-up vertical position for 2 min. Mice were sacrificed 2 days (day 35) after challenge. Lungs were aseptically excised, weighed, and homogenized in 5 mL of sterile peptone water. Homogenates were diluted appropriately, plated in duplicate on blood agar, and incubated for 18 h at 37°C. *S*. *pneumoniae* colonies were counted and results were expressed as log_10_ CFU/g of organ. The presence of bacterial growth in the bloodstream was monitored by obtaining blood samples by cardiac puncture with a heparinized syringe. Samples were plated on blood agar and bacteremia was reported as negative or positive hemocultures after incubation for 18 h at 37 °C.

### Biochemical assay of BAL fluid

Albumin content, a measure to quantitate increased permeability of the bronchoalveolar–capillarity barrier, and lactate dehydrogenase (LDH) activity, an indicator of general cytotoxicity, were determined in BAL of infected animals as described previously [16, 17]. Briefly, albumin content was determined colorimetrically based on albumin binding to bromcresol green using an albumin diagnostic kit (Wiener Lab, Buenos Aires, Argentina). LDH activity, expressed as units per liter of BAL fluid, was determined by measuring the formation of the reduced form of nicotinamide adenine dinucleotide (NAD) using the Wiener reagents and procedures (Wiener Lab).

### Statistical analysis

Each experimental group consisted of 3 mice per group at each time point and experiments were performed in triplicate (n = 9 for each parameter studied). Results were expressed as mean ± standard deviation (SD). The differences between groups were analyzed using student t-test. Differences were considered significant at *p* < 0.05. ANOVA one-way was used for analysis of variance among multiple groups.

## Results

### Nasal immunization with PSPF induce humoral protective immunity

**Fig 1:**
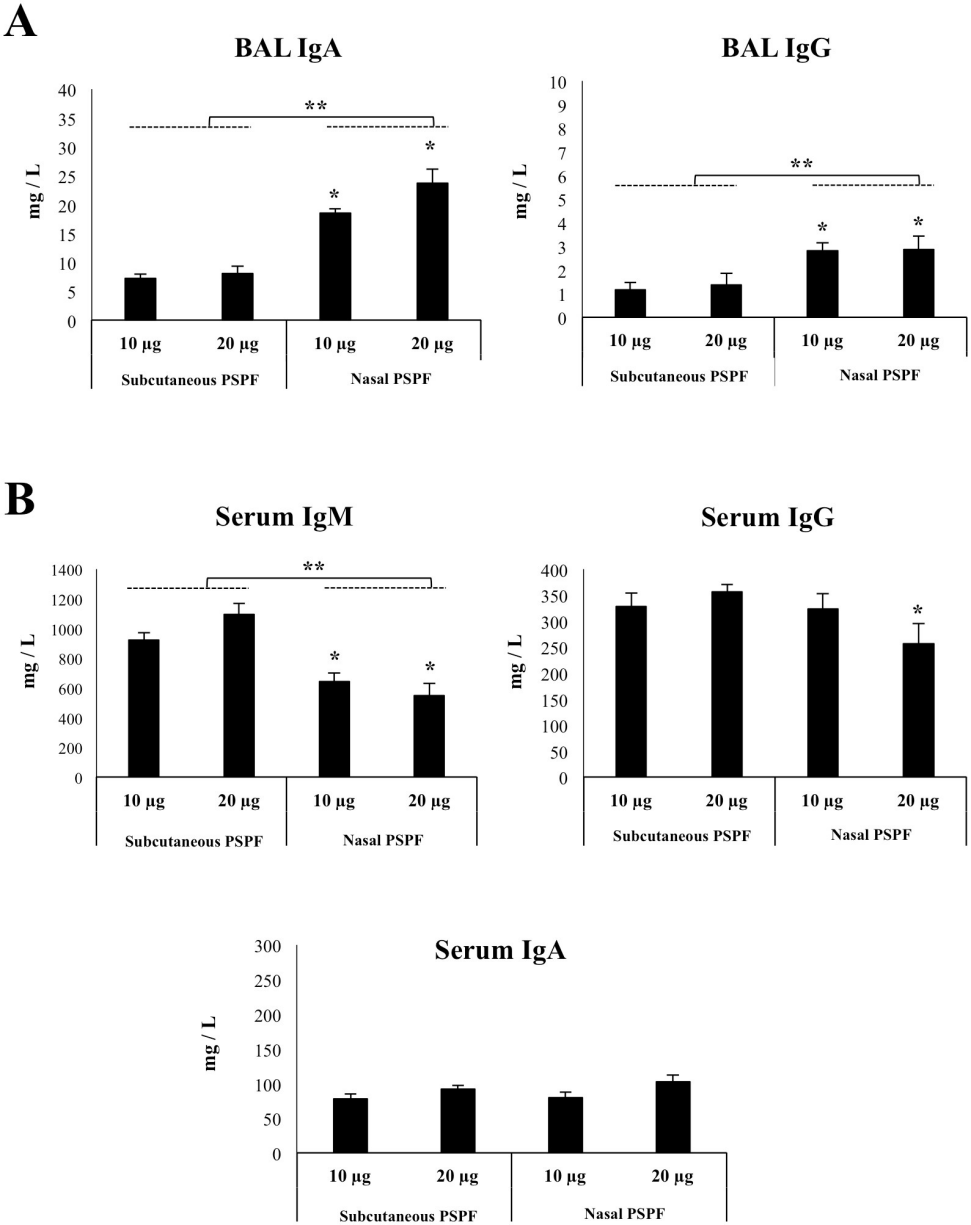
Humoral immune response induced by the immunization with recombinant chimeric pneumococcal protein PSPF (PsaA- Spr1875-PspA-FliC) in infant mice. Swiss albino infant mice (3-week old) received PSPF (10 or 20 μg in 25 μl of sterile PBS) by the nasal route or by subcutaneous injection (10 or 20 μg in 100 μl of sterile PBS) on days 0, 14 and 28. Mice treated by the nasal or subcutaneous routes with sterile PBS were used as controls. Five days after the last immunization, serum and broncho-alveolar lavage (BAL) samples were obtained for the determination of specific antibodies. A) Concentration of PSPF-specific IgA and IgG antibodies in BAL. B) Concentration of PSPF-specific IgM, IgG and IgA in serum. Each experimental group consisted of 3 mice per group and experiments were performed in triplicate (n = 9). Results were expressed as mean ± standard deviation. Differences were considered significant at *p* < 0.05 when compared with animals immunized with the same dose of PSPF (10 or 20 μg) by subcutaneous injection (*) or with the indicated groups (**). Concentrations of specific antibodies in sterile PBS-treated control mice samples were below the detection limit.

We previously demonstrated that the subcutaneous administration of PSPF was able to induce the production of specific IgG antibodies in serum when used in doses between 10 and 20 μg of protein per mouse [10]. Here, we first aimed to evaluate whether the nasal administration of PSPF was effective to induce mucosal and systemic antibodies. For this purpose, infant mice were immunized with 10 or 20 μg of PSPF by the nasal route and subcutaneous immunization was performed in a similar way for comparison. Both specific IgA and IgG antibodies were detected in BAL samples of all the experimental groups (Fig 1). However, levels of BAL IgA and IgG were significantly higher in mice receiving PSPF by the nasal route than those immunized subcutaneously (Fig 1A). The nasal administration of 20 μg of the antigenic protein was able to induce significantly higher levels of BAL IgA when compared with the other groups. Specific IgM, IgG and IgA were also detected in the serum of all the experimental groups (Fig 1B). Subcutaneous immunization was more efficient to induce specific IgM and IgG in serum when compared with the nasal route. No differences were observed between the groups when the levels of serum IgA were compared (Fig 1B).

**Fig 2:**
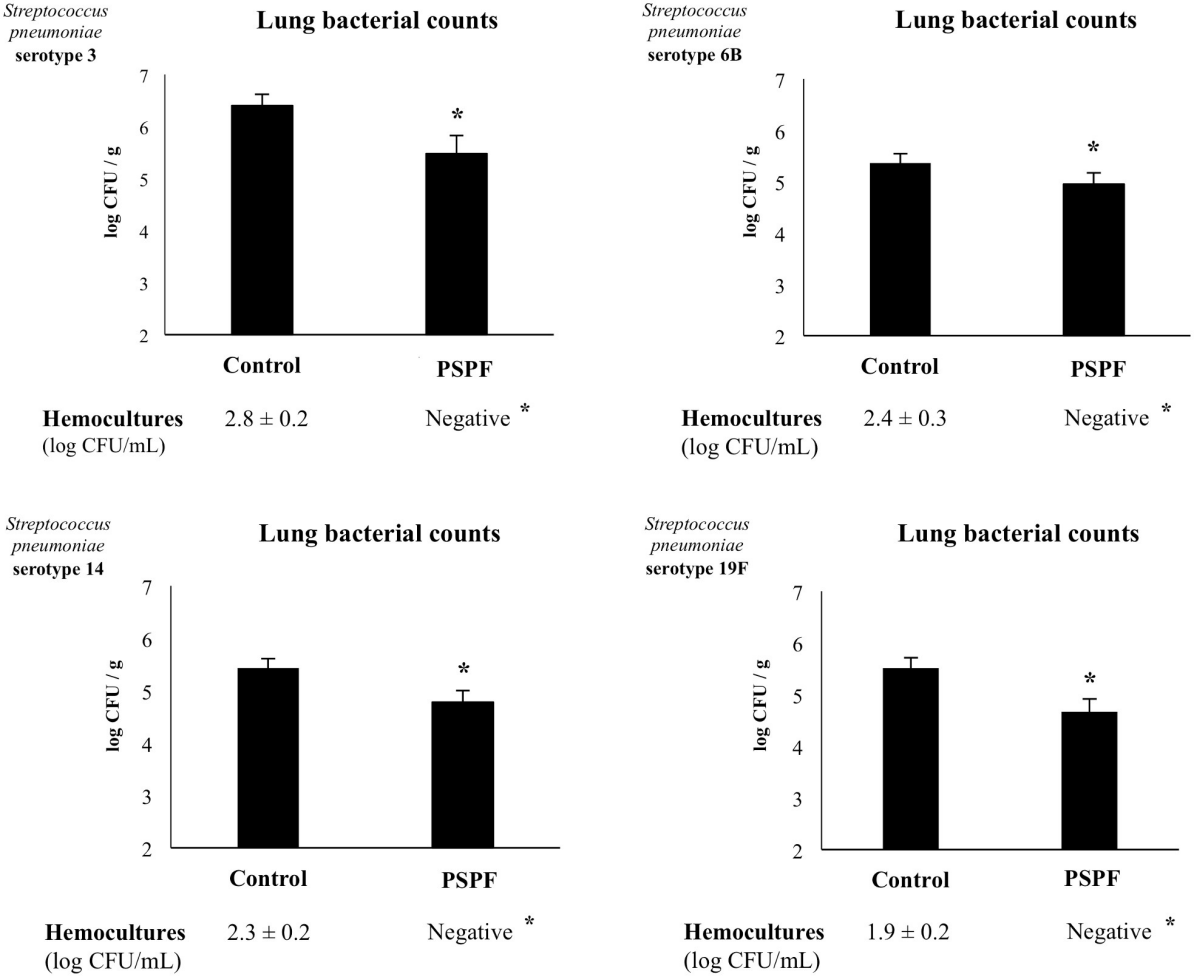
Resistance of infant mice to *Streptococcus pneumoniae* infection induced by the immunization with recombinant chimeric pneumococcal protein PSPF (PsaA- Spr1875-PspA-FliC). Swiss albino infant mice (3-week old) were immunized by nasal route with PSPF (20 μg in 25 μl of sterile PBS) on days 0, 14 and 28. Five days after the last immunization, mice were nasally infected with *S*. *pneumoniae* serotypes 3, 6B, 14, or 19F (10^7^ CFU/mouse). Mice treated by the nasal route with sterile PBS were used as controls. Two days after pneumococcal challenge, lung and blood samples were obtained for the determination of *S*. *pneumoniae* cells counts. Each experimental group consisted of 3 mice per group and experiments were performed in triplicate (n = 9). Results were expressed as mean ± standard deviation. Differences were considered significant at *p* < 0.05 (*) when compared with non-immunized animals.

We next aimed to evaluate whether the humoral immune response induced by the nasal treatment with PSPF was able to confer protection against pneumococcal infection. Infant mice were immunized with 20 μg of PSPF by the nasal route and then infected with different pneumococcal serotypes. As shown in Fig 2, *S*. *pneumoniae* serotypes 3, 6B, 14 and 19F were detected in lung and blood samples of control groups. As we have described previously [12,13], serotype 3 was the most virulent and showed higher counts in lung and blood when compared with serotypes 6B, 14 and 19F. Of note, nasal immunization with PSPF was able to reduce lung bacterial cell counts of the four serotypes evaluated. Moreover, PSPF administration avoided the dissemination of pneumococci into the blood as the hemocultures were negative for the all pneumococcal serotypes (Fig 2).

### Non-viable *L*. *rhamnosus* CRL1505 does not improve the immune response to PSPF

**Fig 3:**
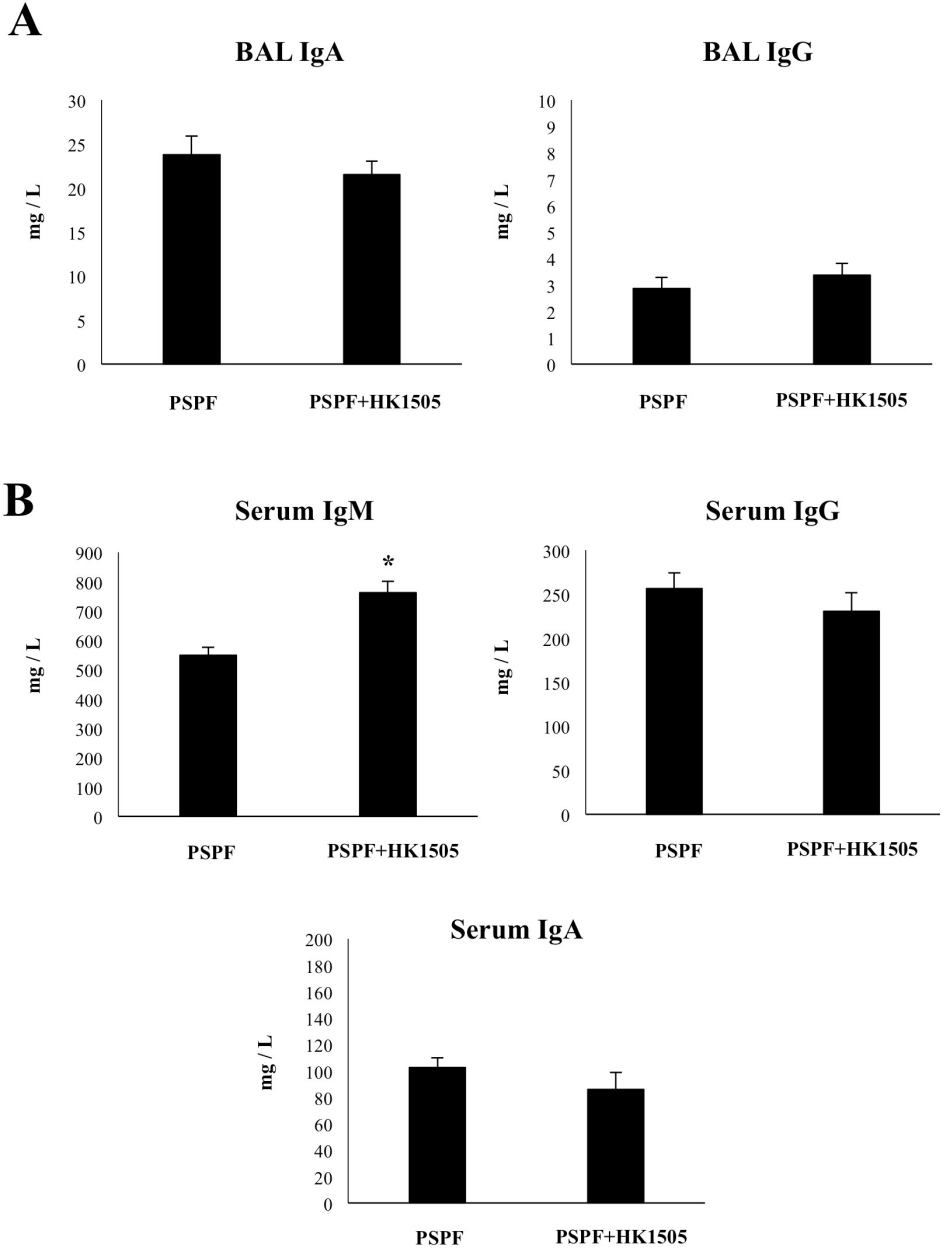
Humoral immune response induced by the immunization with recombinant chimeric pneumococcal protein PSPF (PsaA- Spr1875-PspA-FliC) plus heat-killed *Lactobacillus rhamnosus* CRL1505 in infant mice. Swiss albino infant mice (3-week old) were immunized by nasal route with PSPF (20 μg in 25 μl of sterile PBS) or PSPF (20 μg) plus heat-killed *L*. *rhamnosus* CRL1505 (HK1505) (10^8^ cells) in 25 μl of sterile PBS on days 0, 14 and 28. Five days after the last immunization, serum and broncho-alveolar lavage (BAL) samples were obtained for the determination of specific antibodies. A) Concentration of PSPF-specific IgA and IgG antibodies in BAL. B) Concentration of PSPF-specific IgM, IgG and IgA in serum. Each experimental group consisted of 3 mice per group and experiments were performed in triplicate (n = 9). Results were expressed as mean ± standard deviation. Differences were considered significant at *p* < 0.05 (*) when compared with animals immunized with PSPF only. Concentrations of specific antibodies in sterile PBS-treated control mice samples were below the detection limit.

**Fig 4:**
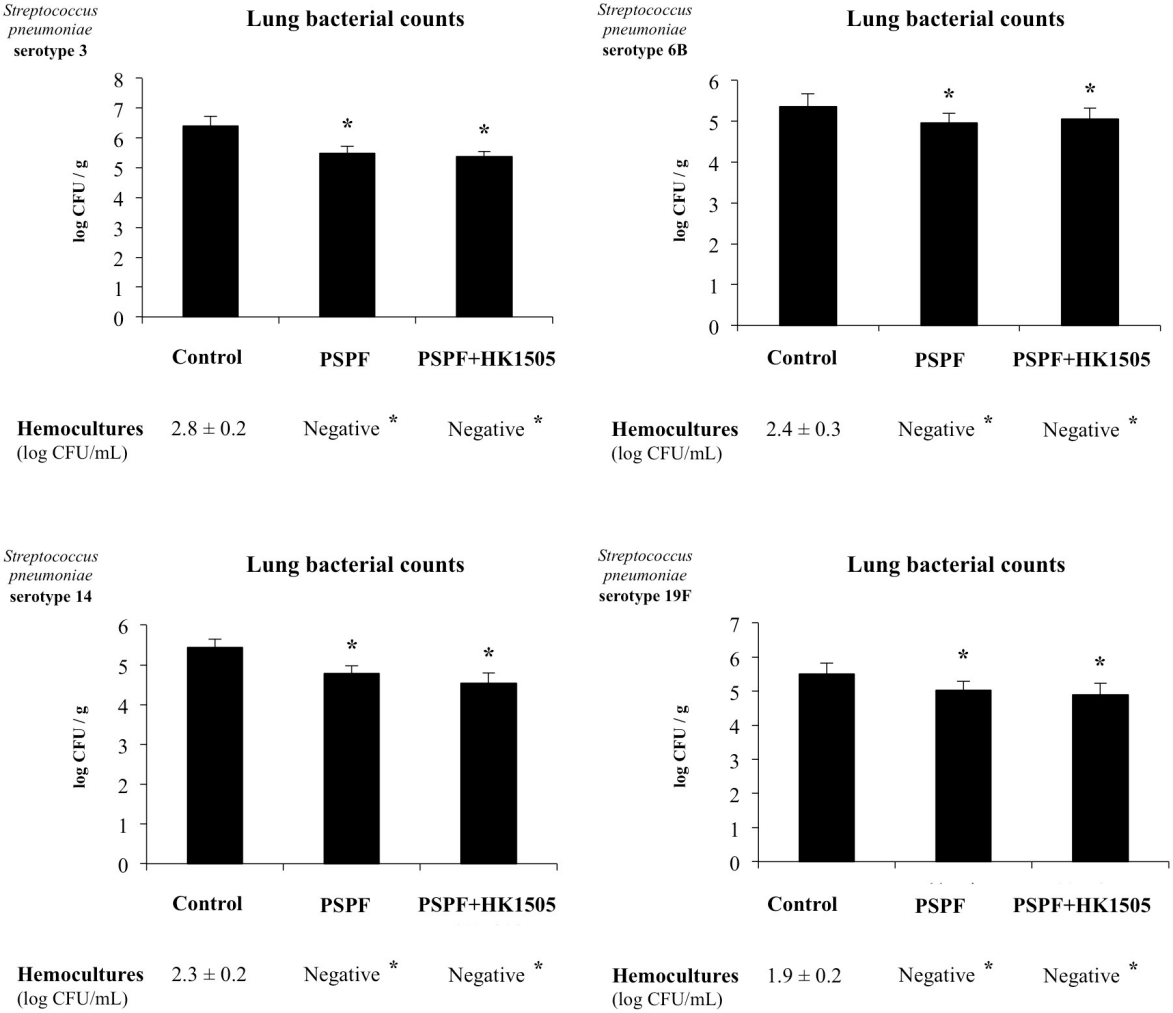
Resistance of infant mice to *Streptococcus pneumoniae* infection induced by the immunization with recombinant chimeric pneumococcal protein PSPF (PsaA- Spr1875-PspA-FliC) plus heat-killed *Lactobacillus rhamnosus* CRL1505. Swiss albino infant mice (3-week old) were immunized by nasal route with PSPF (20 μg in 25 μl of sterile PBS) or PSPF (20 μg) plus heat-killed *L*. *rhamnosus* CRL1505 (HK1505) (10^8^ cells) in 25 μl of sterile PBS on days 0, 14 and 28. Five days after the last immunization, mice were nasally infected with *S*. *pneumoniae* serotype 3, 6B, 14 or 19F (10^7^ CFU/mouse). Two days after pneumococcal challenge, lung and blood samples were obtained for the determination of *S*. *pneumoniae* cells counts. Each experimental group consisted of 3 mice per group and experiments were performed in triplicate (n = 9). Results were expressed as mean ± standard deviation. Differences were considered significant at *p* < 0.05 (*) when compared with non-immunized animals.

We next studied whether the humoral immune response induced by the nasal administration of PSPF could be improved by an additional mucosal adjuvant. Our previous works demonstrated that the nasal administration of viable or non-viable *L*. *rhamnosus* CRL1505 is able to stimulate respiratory and systemic immunity and increase the resistance against respiratory pathogens [15–18]. Then, we performed experiments in which infant mice were immunized with 20 μg of PSPF plus 10^8^ cells of heat-killed *L*. *rhamnosus* CRL1505 (HK1505) by the nasal route (Fig 3). No differences were found in the levels of respiratory and systemic antibodies when PSPF and PSPF+HK1505 groups were compared (Fig 3), with the exception of serum IgM that was higher in the last group. In addition, no differences were observed between both groups when lung bacterial cell counts and hemocultures were compared after the infection with the four pneumococcal serotypes (Fig 4).

### Cell wall from *L*. *rhamnosus* CRL1505 enhances the immune response to PSPF

**Fig 5:**
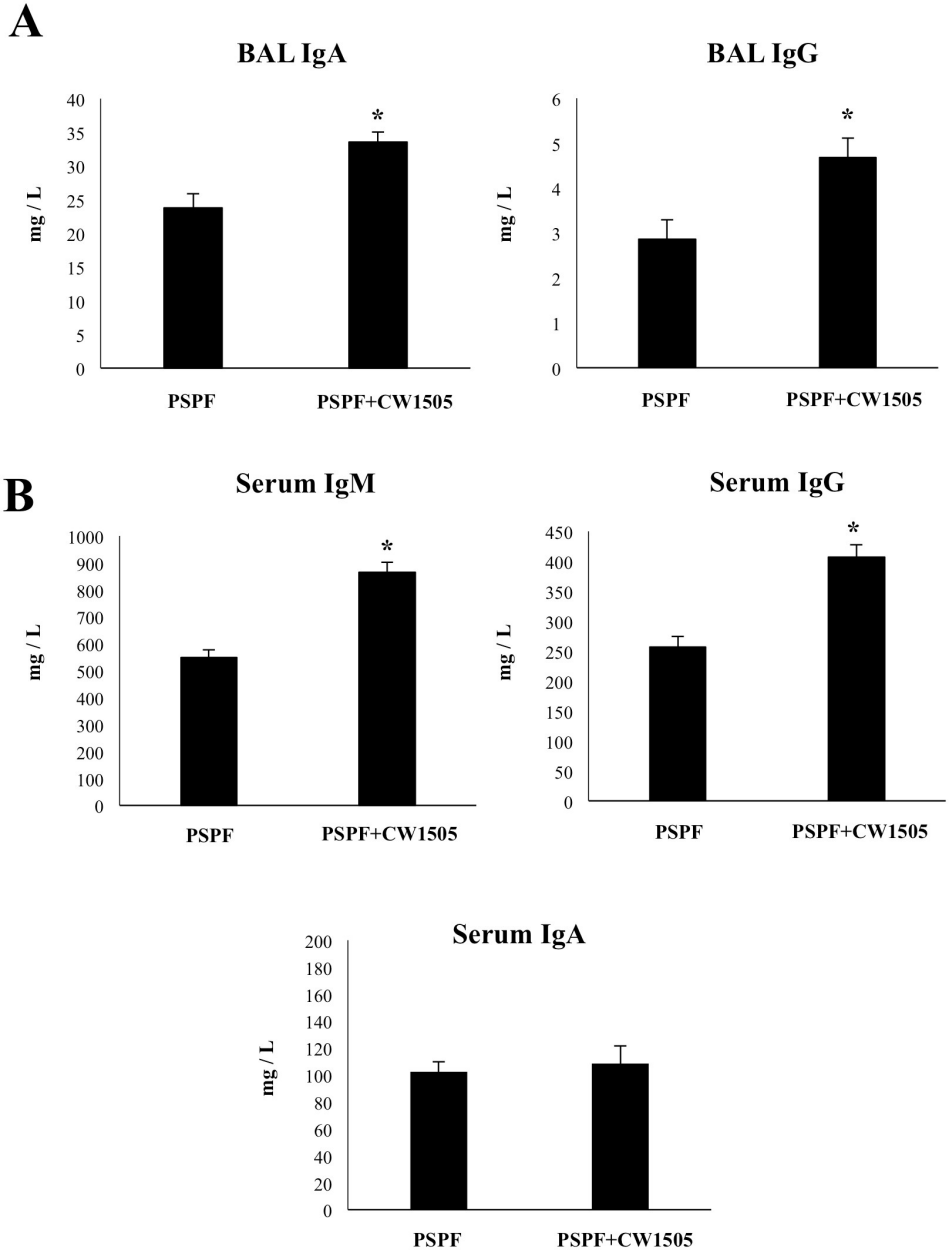
Humoral immune response induced by the immunization with recombinant chimeric pneumococcal protein PSPF (PsaA- Spr1875-PspA-FliC) plus cell wall from *Lactobacillus rhamnosus* CRL1505 in infant mice. Swiss albino infant mice (3-week old) were immunized by nasal route with PSPF (20 μg in 25 μl of sterile PBS) or PSPF (20 μg) plus the cell wall from *L*. *rhamnosus* CRL1505 (CW1505) (8 μg) in 25 μl of sterile PBS on days 0, 14 and 28. Five days after last the immunization, serum and broncho-alveolar lavage (BAL) samples were obtained for the determination of specific antibodies. A) Concentration of PSPF-specific IgA and IgG antibodies in BAL. B) Concentration of PSPF-specific IgM, IgG and IgA in serum. Each experimental group consisted of 3 mice per group and experiments were performed in triplicate (n = 9). Results were expressed as mean ± standard deviation. Differences were considered significant at *p* < 0.05 (*) when compared with animals immunized with PSPF only. Concentrations of specific antibodies in sterile PBS-treated control mice samples were below the detection limit.

We have previously demonstrated that some cellular fractions of *L*. *rhamnosus* CRL1505 such as its cell wall have immunomodulatory activities [15, 18]. Then, we next aimed to evaluate whether the cell wall of *L*. *rhamnosus* CRL1505 (CW1505) would be a more suitable mucosal adjuvant than non-viable bacteria. For this purpose, infant mice were nasally immunized with 20 μg of PSPF with or without the addition of 8 μg of CW1505 and the humoral immune response was evaluated (Fig 5). The immunization of infant mice with PSPF+CW1505 significantly increased the levels of BAL IgA and IgG when compared with animals that received only PSPF (Fig 5). No differences between the groups were found when serum IgA was analyzed while serum IgM and IgG were significantly higher in mice treated with PSPF+CW1505 than PSPF alone (Fig 5).

**Fig 6:**
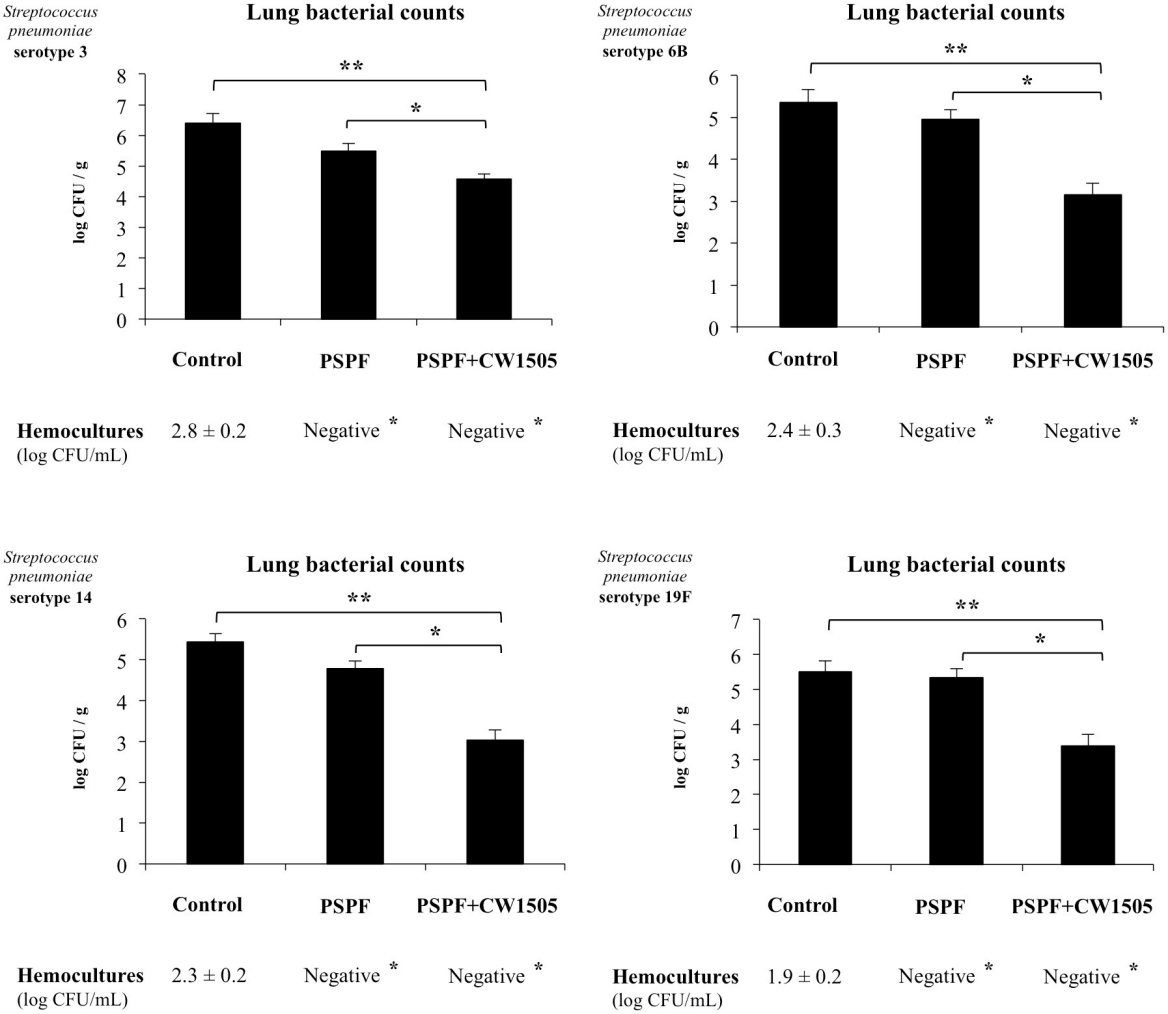
Resistance of infant mice to *Streptococcus pneumoniae* infection induced by the immunization with recombinant chimeric pneumococcal protein PSPF (PsaA- Spr1875-PspA-FliC) plus cell wall from *Lactobacillus rhamnosus* CRL1505. Swiss albino infant mice (3-week old) were immunized by nasal route with PSPF (20 μg in 25 μl of sterile PBS) or PSPF (20 μg) plus cell wall from *L*. *rhamnosus* CRL1505 (CW1505) (8 μg) in 25 μl of sterile PBS on days 0, 14 and 28. Five days after the last immunization, mice were nasally infected with *S*. *pneumoniae* serotype 3, 6B, 14 or 19F (10^7^ CFU/mouse). Two days after pneumococcal challenge, lung and blood samples were obtained for the determination of *S*. *pneumoniae* cells counts. Each experimental group consisted of 3 mice per group and experiments were performed in triplicate (n = 9). Results were expressed as mean ± standard deviation. Differences in bacterial cell counts in lungs were considered significant at *p* < 0.05 when compared with animals immunized with PSPF only (*) or non-immunized mice (**). Differences in hemocultures were considered significant at *p* < 0.05 (*) when compared with non-immunized animals.

**Fig 7:**
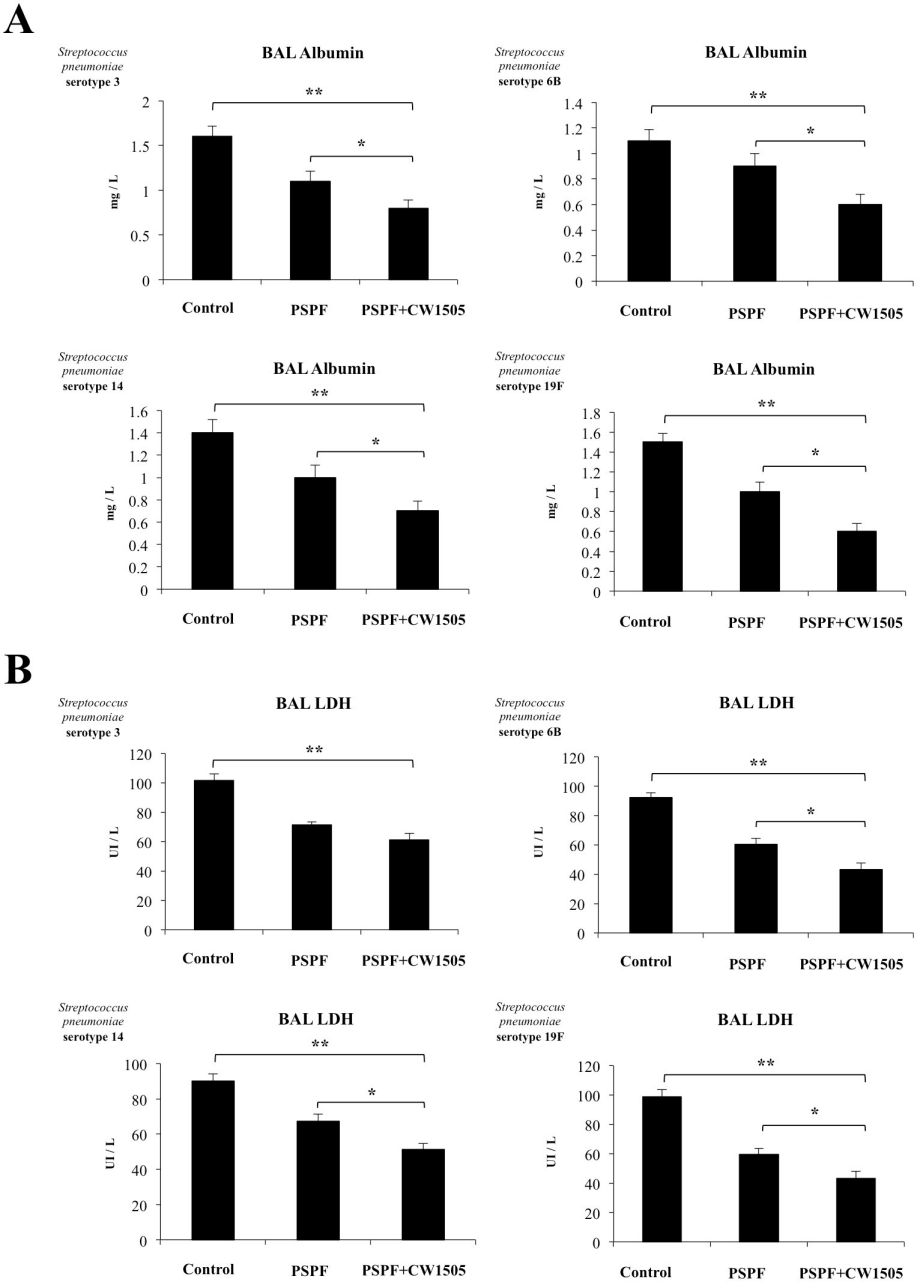
Lung injuries in infant mice immunized with the recombinant chimeric pneumococcal protein PSPF (PsaA- Spr1875-PspA-FliC) plus cell wall from *Lactobacillus rhamnosus* CRL1505 and infected with *Streptococcus pneumoniae*. Swiss albino infant mice (3-week old) were immunized by nasal route with PSPF (20 μg in 25 μl of sterile PBS) or PSPF (20 μg) plus cell wall from *L*. *rhamnosus* CRL1505 (CW1505) (8 μg) in 25 μl of sterile PBS on days 0, 14 and 28. Five days after the last immunization, mice were nasally infected with *S*. *pneumoniae* serotype 3, 6B, 14 or 19F (10^7^ CFU/mouse). Two days after pneumococcal challenge, broncho-alveolar lavage (BAL) samples were obtained for the determination of albumin concentrations and lactate dehydrogenase (LDH) activity. Each experimental group consisted of 3 mice per group and experiments were performed in triplicate (n = 9). Results were expressed as mean ± standard deviation. Differences in BAL biochemical parameters were considered significant at *p* < 0.05 when compared with animals immunized with PSPF only (*) or non-immunized mice (**).

Similar to PSPF immunization, hemocultures of mice receiving PSPF+CW1505 were negative for all the pneumococcal serotypes analyzed (Fig 6). In addition, lung bacterial cell counts were significantly lower in PSPF+CW1505 than in animals immunized with PSPF alone (Fig 6). In order to evaluate whether immunization with PSPF or PSPF+CW1505 protected infant mice against lung damage, albumin content and LDH activity were evaluated in BAL samples, as measures of alveolar-capillary barrier alteration and cellular damage, respectively (Fig 7). All the pneumococcal serotypes increased the levels of albumin (Fig 7A) and LDH (Fig 7B) in BAL of infant mice. Both, PSPF or PSPF+CW1505 treatments were able to significantly reduce these biochemical parameters in the BAL of infected infant mice indicating lower lung damage. However, the ability of PSPF+CW1505 to reduce lung damage was significantly higher when compared to the observed for PSPF immunization (Fig 7).

### Non-viable *L*. *rhamnosus* CRL1505 and its cell wall induce a different cytokine profile

**Fig 8:**
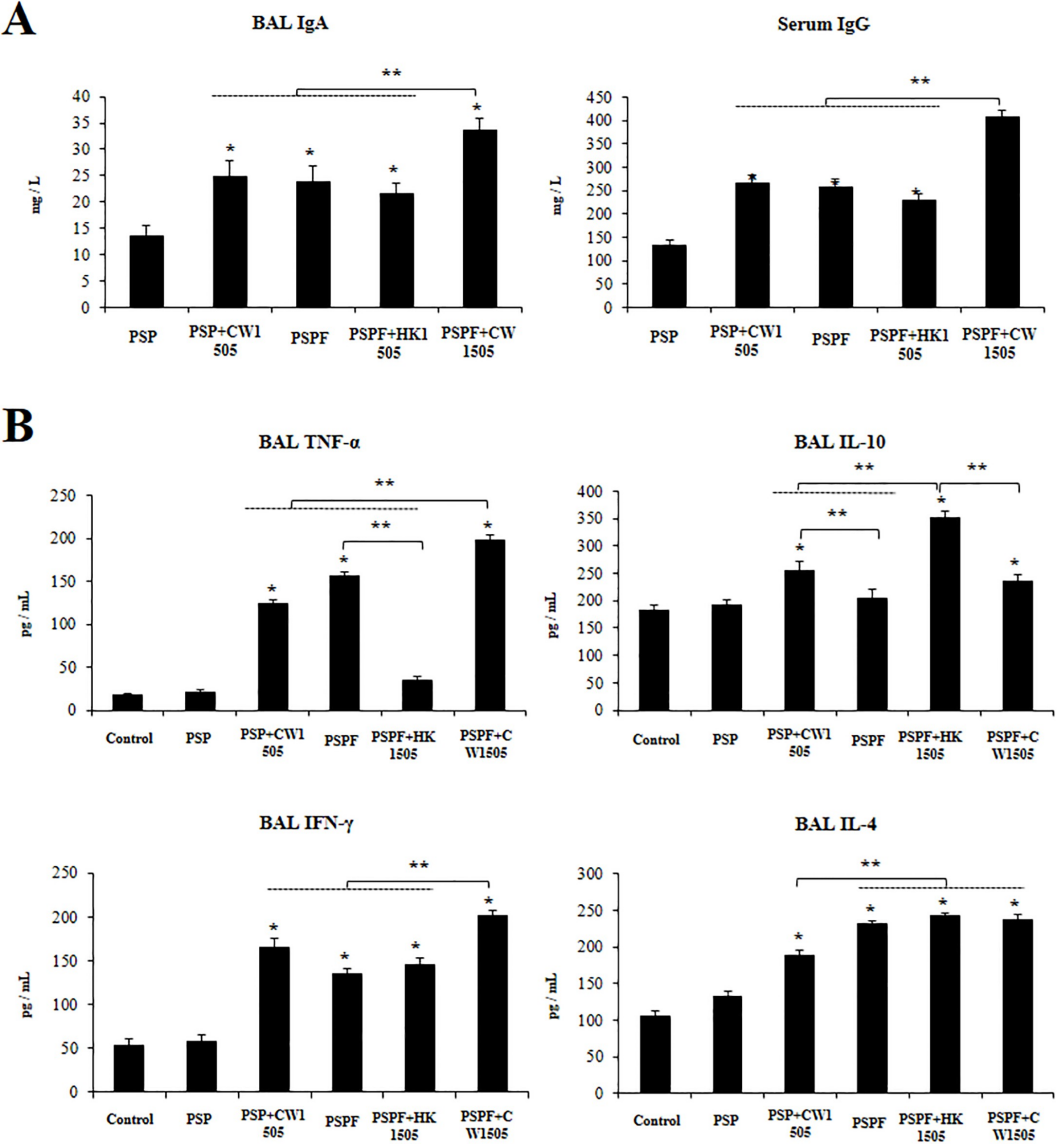
Immune response induced by the immunization with recombinant chimeric pneumococcal protein PSPF (PsaA- Spr1875-PspA-FliC) or PSP (PsaA- Spr1875-PspA) plus non-viable *Lactobacillus rhamnosus* CRL1505 or its cell wall in infant mice. Swiss albino infant mice (3-week old) were immunized by nasal route with PSPF or PSP (20 μg in 25 μl of sterile PBS), PSPF (20 μg) plus heat-killed *L*. *rhamnosus* CRL1505 (HK1505) (10^8^ cells), or PSPF or PSP (20 μg) plus the cell wall from *L*. *rhamnosus* CRL1505 (CW1505) (8 μg) in 25 μl of sterile PBS on days 0, 14 and 28. A) Five days after the last immunization, serum and broncho-alveolar lavage (BAL) samples were obtained for the determination of IgA and IgG specific antibodies. Concentrations of specific antibodies in sterile PBS-treated control mice samples were below the detection limit. B) One day after the last immunization BAL samples were obtained for the determination of tumour necrosis factor (TNF)-α, interferon (IFN)-γ, interleukin (IL)-4, and IL-10 concentrations. Each experimental group consisted of 3 mice per group and experiments were performed in triplicate (n = 9). Results were expressed as mean ± standard deviation. Differences were considered significant at *p* < 0.05 when compared with animals immunized with PSP only (*) or with the indicated groups (**).

Finally, we performed a final set of experiments in which infant mice were immunized with PSPF, PSPF+HK1505 or PSPF+CW1505 and two additional groups in which animals were immunized with the recombinant chimeric pneumococcal protein lacking the FliC fragments: PSP and PSP+CW1505 groups (Fig 8). Immunization with PSP induced the production of specific BAL IgA and serum IgG antibodies (Fig 8A). However, the levels of both antibodies were significantly lower when compared to the values observed in mice receiving PSPF. Interestingly, administration of PSP with CW1505 increased BAL IgA and serum IgG antibodies that reached the levels found in the PSPF group (Fig 8A). Of note, the levels of respiratory and blood antibodies were significantly higher in PSPF+CW1505 when compared to all the other experimental groups (Fig 8A).

The levels of respiratory TNF-α, IFN-γ, IL-4, and IL-10 were evaluated one day after the last immunization (day 29). Administration of PSP did not change the levels of the evaluated cytokines when compared to untreated control infant mice (Fig 8B). PSPF increased the levels of BAL TNF-α, IFN-γ, and IL-4 while no effect was observed for IL-10. Interestingly, mice immunized with PSP+CW1505 had levels of BAL TNF-α, and IFN-γ that were not statistically different from those found in the PSPF group (Fig 8B). Of note, BAL IL-10 and IL-4 were higher in PSP+CW1505 mice when compared to PSPF animals (Fig 8B). Mice immunized with PSPF+HK1505 showed improved levels of IL-10 when compared to PSPF group while their levels of BAL TNF-α were not different from PSP or untreated control infant mice (Fig 8B). In addition, the PSPF+HK1505 group had significantly higher levels of BAL IL-10 when compared to PSP+CW1505 mice. The levels of respiratory TNF-α and IFN-γ were significantly higher in PSPF+CW1505 mice when compared to all the other experimental groups (Fig 8B).

## Discussion

We have reported previously that systemic immunization of immunocompetent adult mice with the recombinant chimeric pneumococcal protein PSPF induced specific humoral immune responses and significantly reduced the susceptibility to pneumococcal infection [10]. In contrast with systemic immunization, mucosal administered vaccines have the distinct advantage of being able to reach the highly concentrated mucosal associated lymphoid tissues, and induce both systemic and mucosal immune responses [19]. In this work, we demonstrated that the nasal immunization of infant mice with PSPF was able to improve the resistance to the respiratory pathogen *S*. *pneumoniae* in lung colonization assays. These findings are of note because infant mice are more susceptible to pneumococcal infection when compared to adult animals [12,13].

Challenge experiments with *S*. *pneumoniae* belonging to serotypes 3, 6B, 14 and 19F were performed in infant mice in order to evaluate the efficacy of the immunization with PSPF against the respiratory infection produced with different pneumococcal serotypes. As we have reported previously [12,13], non-immunized infant mice can be successfully infected with the four pneumococcal serotypes. The respiratory pathogen was detected in lung and blood samples indicating that it is able to colonize the respiratory mucosa and disseminate into the blood. Moreover, the levels of BAL albumin and LDH in infected infant mice were significantly higher compared to non-infected controls indicating that infection altered the permeability of the bronchoalveolar–capillarity barrier and induced cytotoxicity [12,13]. However, immunization with PSPF significantly reduced lung colonization, provided protection against bloodstream dissemination and diminished lung injuries for all the serotypes evaluated.

The nasal immunization with PSPF induced the production of both specific IgA and IgG antibodies in the respiratory tract of infant mice. As expected, the levels of both antibodies were significantly higher in nasally primed mice when compared to those immunized by the subcutaneous route. It is well known that the efficient protection of the respiratory tract depends of both IgA and IgG antibodies. The airways are mainly protected by IgA responses while in the deep lung the production of IgG is of importance for the defence against pathogens [20]. Thus, the production of both types of antibodies could be involved in the improved resistance of infant mice nasally immunized with PSPF against pneumococcal lung colonization when compared with those vaccinated by the subcutaneous route.

Therefore, we have advanced in the characterization of the PSPF experimental vaccine by evaluating several important factors that have to be taken into account when developing a pneumococcal vaccine: it is immunogenic when administered through the nasal route, it is effective in young hosts, and protects against different serotypes of the respiratory pathogen.

An important obstacle for the development of mucosal vaccines is the weak induction of immune responses triggered by antigens administered without the addition of mucosal adjuvants. Therefore, the search for effective and safe mucosal adjuvants becomes imperative. The results of our previous work [10], and those presented here indicated that the flagellin fragments included in the chimeric PSPF molecule acted as an efficient mucosal adjuvant, probably through their interaction with TLR5. In fact, immunization of infant mice with PSP, the chimeric pneumococcal protein lacking the flagellin fragments, had significantly lower levels of respiratory and serum specific antibodies when compared to mice receiving the PSPF. Moreover, PSP was less efficient to increase the levels of respiratory TNF-α and IFN-γ than PSPF. In line with our findings, it was demonstrated that the nasal administration of flagellin stimulated T-cell-mediated immunity, and improved the production of respiratory and systemic antibodies against model antigens in a TLR5-dependent manner [21]. Moreover, it was reported that flagellin triggered a TLR5-mediated signalling in airway epithelium and induced the production of stimulatory factors such as TNF-α and IFN-γ for lung dendritic cells. Stimulated mature lung antigen presenting cells were involved in the enhanced cellular and humoral immune responses [22].

Despite the efficiency of the flagellin domains contained in the PSPF molecule to function as mucosal adjuvant, we wanted to investigate whether the co-administration of this experimental vaccine with other mucosal adjuvants could further enhance the specific humoral immune response and the protection against pneumococci.

Beneficial microbes have been tested as potential adjuvants, since they are considered safe microorganisms, and some of them have been proved able to modulate the mucosal immune system [23,24]. In this regard, research work has demonstrated that nasal administration of *L*. *rhamnosus* CRL1505 is an interesting alternative to improve immunity in the respiratory tract and confer protection against pathogens like *S*. *pneumoniae* [25], Respiratory Syncytial Virus [26] or Influenza Virus [27]. Moreover, we have previously shown that non-viable *L*. *rhamnosus* CRL1505, its cell wall or purified peptidoglycan are also able to beneficially influence respiratory immune responses when nasally administered [15,18,26].

In this work, we observed that the co-administration of non-viable *L*. *rhamnosus* CRL1505 with PSPF did not induce changes in the humoral immune response of infant mice. On the contrary, when PSPF was administered to mice together with the cell wall of the CRL1505 strain a significant improvement in the induction of specific immune response and the protection against pneumococci was detected. The differences in the adjuvant capacity of non-viable immunobiotic and its cell wall could be related to the different profile of cytokines induced after their contact with cells of the respiratory mucosa. Similarly to viable *L*. *rhamnosus* CRL1505, we have reported previously that non-viable lactobacilli are able to interact with epithelial cells and antigen presenting cells such as alveolar macrophages and induce the production of both pro-inflammatory cytokines as well as IL-10 in the respiratory tract [15, 26]. In line with these findings, we observed here that nasal immunization of infant mice with PSPF+HK1505 improved the respiratory levels of IFN-γ and IL-4 but not TNF-α. Moreover, PSPF+HK1505 treatment was the most efficient to increase IL-10 in the respiratory tract. Therefore, the TNF-α/IL-10 ratio induced by HK1505 would be not optimal to stimulate the activation of antigen presenting cells and induce a specific response to PSPF.

On the other hand, CW1505 improved the levels of all the cytokines studied including TNF-α, IFN-γ and IL-4 that are able to stimulate antigen presentation. In addition, the TNF-α/IL-10 ratio induced by CW1505 was higher than the observed for HK1505. This is in agreement with our previous findings demonstrating that the nasal administration of *L*. *rhamnosus* CRL1505 cell wall significantly increased the production of TNF-α, IL-1β and IL-6 in the respiratory tract [15]. As other Gram-positive bacteria, *L*. *rhamnosus* CRL1505 cell wall is comprised by a complex mixture of glycolipids, lipoproteins, and phosphorylated polysaccharides embedded in a thick layer of peptidoglycan. It is well known that several of these components act as microbe-associated molecular patterns that are recognized by different pattern-recognition receptors expressed in epithelial and antigen presenting cells including TLR2 and nucleotide-binding oligomerization domain (NOD) receptors [28]. Activation of TLR2 and NOD receptors finally trigger the production of pro-inflammatory cytokines such as TNF-α, IL-1β and IL-6 that participate in the maturation and activation of antigen presenting cells. Therefore, activation of receptors like TLR2 and/or NOD in the respiratory tract could be involved in the potentiation of the immune response to PSPF induced by the cell wall of the immunobiotic lactobacilli. An interesting topic for future research would be to determine the cells and receptors that are activated by *L*. *rhamnosus* CRL1505 cell wall that are related to the improvement of antigen presentation. In addition, considering that the capacity of lactobacilli to stimulate the immune system is a strain specific property [28,29], it would be important to evaluate whether the cell wall of CRL1505 strain has unique immunomodulatory properties as vaccine adjuvant or if this property is common to other immunobiotic strains.

In summary, the results presented in this work shown that the nasal immunization with PSPF elicited cross-protective immunity against different pneumococcal serotypes, reduced the susceptibility of infant mice to the respiratory infection evaluated in lung colonization assay, and diminished lung injuries. Our results also suggest that immunization with PSPF together with the cell wall of the immunobiotic strain *L*. *rhamnosus* CRL1505 as a second mucosal adjuvant significantly increased the effectiveness of the experimental vaccine. The results of this work are of importance since preclinical studies in animal models and human clinical trials have reported that the early life immunization may result in efficient priming that can provide an excellent basis for future boosting [30]. Therefore, early immunization with PSPF and cell wall from *L*. *rhamnosus* CRL1505 could be an interesting alternative to improve protection against pneumococcal infection in children and adults.
